# Risk assessment of acute kidney injury following cardiopulmonary bypass

**DOI:** 10.1186/s13019-020-01382-x

**Published:** 2021-01-06

**Authors:** Thomas Wittlinger, Martin Maus, Ingo Kutschka, Hassina Baraki, Martin G. Friedrich

**Affiliations:** 1 Department of Cardiology, Asklepios Hospital Goslar, Köslinerstr 12, 38642 Goslar, Germany; 2 Department Major Surgery, St. Elisabeth Hospital, Bonn, Germany; 3 Department of Cardio-Thoracic and Vascular Surgery, University Hospital Göttingen, Göttingen, Germany

**Keywords:** Acute kidney injury, Extracorporeal circulation, RIFLE classification, Continuous veno-venous hemodialysis, Cardiac surgery

## Abstract

**Background:**

Acute kidney injury (AKI) is a frequent and serious complication of cardiac surgery, associated with a high incidence of morbidity and mortality. Although the RIFLE criteria serve as a prominent tool to identify patients at high risk of AKI, an optimized diagnosis model in clinical practice is desired.

**Methods:**

Based on the SOP-criteria, 365 patients (10%) developed AKI following surgery and were subjected to RRT. In contrast, the incidence of AKI, defined according to the RIFLE criteria, was only 7% (*n* = 251 patients). Prominent risk factors identified by SOP were patients’ sex, valve and combined valve and bypass surgery, deep hypothermia, use of intra-aortic balloon pump (IABP) and previous coronary interventions. Ischemia, reperfusion, blood loss and surgery time also served as significant risk factors for patient evaluated by SOP.

**Results:**

Risk assessment by RIFLE differed in as much as most patients with normothermia and those receiving only cardiovascular bypass developed AKI. However, patients’ sex and valve surgery did not serve as a risk factor.

**Conclusion:**

Evaluation of patients by the RIFLE versus SOP criteria yielded different results with more AKI patients detected by SOP. Based on the present data, it is concluded that patients may not prone to AKI when surgery and ischemia time will be kept short, when blood loss is mitigated to a minimum and when surgery is performed under non-hypothermic conditions.

## Introduction

The development of the heart-lung machine has revolutionized cardiac surgery. Still, cardiovascular surgery is associated with severe complications, whereby acute kidney injury (AKI) has been particularly linked with a high incidence of morbidity and mortality [1]. 2–5% of patients developing AKI after cardiac surgery require renal replacement therapy (RRT) that may further increase mortality [2].

The percentage of the AKI rates vary depending on the study populations and the AKI definitions. Hu and colleagues using either the RIFLE (Risk, Injury, Failure, Loss, End-Stage Renal Disease, based on serum creatinine, urine output and/or glomerular filtration rate, GFR), AKIN (Acute Kidney Injury Network, based on serum creatinine and not on GFR changes), or KDIGO (Kidney Disease: Improving Global Outcomes) criteria (combining the differences between the RIFLE and AKIN) have estimated a global incidence of AKI after cardiac surgery at 22.3% [1]. Based on the pRIFLE (Pediatric Risk, Injury, Failure, Loss, End Stage Renal Disease) criteria, AKI developed postoperatively in 24.9% of the patients [3], and defining AKI by the KDIGO criteria revealed that 42% of the patients included in the respective study met the diagnostic criteria for AKI [4].

The aetiology of AKI is rather complex and not evaluated in detail. Evans et al. concluded that renal haemodynamics and oxygenation during and after cardiac surgery may (at least partially) trigger AKI [5], and a meta-analysis determined hypertension, alterations of serum creatinine, cardiopulmonary bypass time, aortic clamping time, use of an intra-aortic balloon pump (among others) as strong predictors for the development of AKI postoperatively [6].

In this matter, Jiang et al. criticized that actually no consensus AKI definition has been used in the risk scores established, with the consequence that none of these models may accurately predict the incidence of AKI [7]. In fact, based on a meta-analysis dealing with the utility of serum biomarkers, discrimination capability for cardiac surgery–associated AKI in the early postoperative period was only modest. Even the control of “established” functional biomarkers such as urine output and serum creatinine may allow only a delayed diagnosis which is inadequate in an acute setting.

To guarantee optimal patient guiding and intervention, the relevant risk factors for AKI have to be identified to develop highly efficient prediction tools for use in routine clinical practice. In the current investigation, the incidence of AKI of 3574 patients following cardiac surgery in a German hospital has been calculated by the RIFLE versus hospital own criteria.

## Methods

The present retrospective study was approved by the local ethics committee of the University Hospital of Göttingen, Germany (No.16–4-10) and included 3574 patients (mean age 66,5 years) who underwent open heart surgery requiring extracorporeal circulation. Data were collected from January 2000 to December 2005 using the GISI and GISI-OP database from the Göttingen University Hospital. Kind of surgery included isolated bypass grafting, isolated aortic or mitral valve reparation/replacement, both combined bypass grafting and valve surgery or neither bypass nor heart valve operations. Patients below 18 years were excluded from the study.

### Acute kidney injury

Patients were divided into two groups, cohort 1 with normal renal function post-surgery and cohort 2 who developed acute kidney injury (AKI) and was subjected to renal replacement therapy (RRT) done by continuous veno-venous hemodialysis (CVVH; multiFiltrate, Fresenius Medical Care, Bad Homburg, Germany). Assessment of renal function after surgery was done by two different criteria: Criterion 1 was based on the “Standard Operating Procedure” (SOP), defined by the Centre for Anaesthesiology, Emergency and Intensive Care Medicine, Göttingen University Hospital:

- Pulmonary oedema or impending right ventricular decompensation due to overhydration which cannot be treated by diuretics,
- Uraemic complications (encephalopathy, neuropathy, pericarditis, acidosis),
- Hyperkalemia (> 6,5 mmol/l).

Criterion 2 was based on the RIFLE classification, with a threefold elevation of serum creatinine, or a maximum creatinine level of 4.0 mg/dl, or a decrease of the glomerular filtration rate (GFR) by 75%, compared to the preoperative value. This analysis was consistent with the R, I and F stage of the RIFLE criteria.

### Serum creatinine

Serum creatinine level (mg/dl) has been estimated using the Cockcroft-Gault equation. The equation includes the variables: age, gender, body weight and glomerular filtration rate (GFR; mL/min/1.73 m^2^) and reads: GFR = [(150 – age) x body weight (kg) / serum creatinine (μmol/l)] – gender factor (10% males, 15% females).

### Statistics

Statistical analysis was performed with Statistica software version 7.0 (StatSoft Europe GmbH, Berikon, Switzerland) for most of the data sets.

Metric variables of interest were patient age, serum creatinine level (evaluated immediately after surgery, “creatinine A”, or 24 h after surgery, “creatinine B”), duration of surgery, duration of ischemia, (re) perfusion time, bleeding (blood loss), body temperature.

Categorical parameters included gender, type of surgical intervention, use of an intra-aortic balloon pump (IABP), centrifugal versus roller blood pump, cardioplegia (blood cardioplegia versus Bretschneider), intraoperative body temperature (normothermia: 37–34 °C, mild hypothermia: 33–28 °C, deep hypothermia: 27–18 °C).

Non parametric Kruskal-Wallis test was used for the longitudinal analysis of blood loss, and Wilcoxon-Mann-Whitney-test was applied for comparing the metric variables. Relationship between serum creatinine and the categorical parameters was evaluated by the Wilcoxon-test or the Kruskal-Wallis test. Association between creatinine level and the metric parameters was analyzed by the Pearson correlation coefficient ρ. Categorical proportions were compared using the chi-squared test. All results were considered to be significant at α = 5% (*p* <  0.05).

## Results

**Fig. 1:**
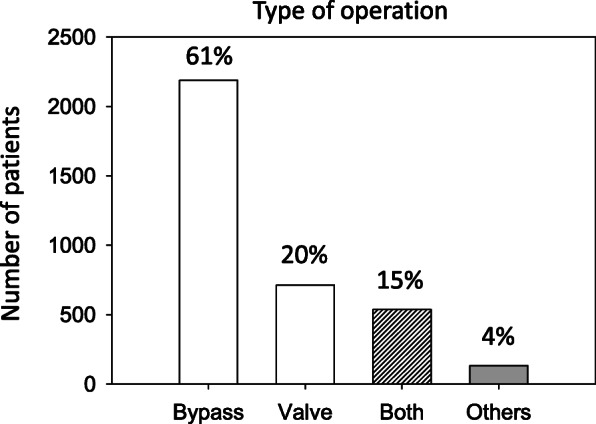
Type of surgical procedure

Proportion of male patients (2522; 71.0%) dominated over females (1052; 29%). The median age of the patient cohort was 68 years (min.: 18 years; max.: 92 years) with a 25%-quantile of 61 and a 75%-quantile of 74 years. Mean age of the 2522 male and 1052 female patients was 63,5 years and 69,25 years. Operative characteristics are shown in Fig. 1. The majority of patients received coronary artery bypass grafts (61%). Isolated aortic valve replacement was performed in 20% of the patients, and 15% of the patients had aortic valve replacement which was combined with coronary surgery. Overall mortality was 6.4% (*n* = 230).

### Acute kidney injury defined by SOP

Based on the SOP-criteria, 365 patients (10%) developed AKI following surgery and were subjected to hemofiltration. One hundred seventy-seven patients died within a mean of 12 +/− 16.69 days (min.: day 0 (day of surgery), max.: day 130) which corresponded to a mortality rate of this patient group of 48%.

The incidence of AKI and CVVH was significantly influenced by the patients’ sex, the type of surgery, and intraoperative temperature (Table 1). Significantly more females developed AKI and received hemofiltration than males did. Cardiac valve surgery and combined valve and bypass surgery, but not bypass grafting alone, also served as significant predictors of AKI. Interestingly, operations apart from valve and bypass surgery („others“) have been associated with a greater risk to develop AKI as well. This was also true with respect to previous coronary interventions, where patients developed AKI more often than patients undergoing first surgery. Use of intra-aortic balloon pump (IABP) also served as an independent risk factor for AKI. Finally, AKI and hemofiltration occured significantly more often in patients with deep hypothermia, compared to mild or normothermia.

**Table 1:** Demographic and clinical parameters in the acute kidney injury (AKI) group according to the SOP-classification

Parameter	RRT		*p*-value (chi-squared test)
	No	Yes	
	*n* = 3209	*n* = 365	
Sex			
Male	2288 (91%)	234 (9%)	
Female	920 (88%)	131 (12%)	< 0.01
Valve surgery			
Yes	1090 (87%)	161 (13%)	
No	2119 (91%)	204 (9%)	< 0.01
Temperature			
Normothermia	1789 (92%)	163 (8%)	
Mild hypothermia	693 (87%)	100 (13%)	
Deep hypothermia	14 (74%)	5 (26%)	< 0.01
Surgery			
Bypass	2006 (92%)	184 (8%)	
Valve	645 (90%)	69 (10%)	
Bypass+valve	445 (83%)	92 (17%)	< 0.01

Metric parameters were also evaluated and are depicted in Table 2. All variables, age, creatinine level post operation, operation duration, ischemia time, minimum intraoperative body temperature, perfusion and reperfusion time, correlated positively with the development of AKI, necessitating RRT.

**Table 2:** Influence of metric variables on renal replacement therapy according to the SOP-classification

Parameter	RRT*						*p*-value (Wilcoxon)
	No			Yes			
	*n* = 3209			*n* = 365			
	25%	50%	75%	25%	50%	75%	
Age (years)	61.0	68.0	74.0	66.0	71.0	76.0	< 0.01
Creatinine A (mg/dl)	0.8	0.9	1,1	1.1	1.5	2.0	< 0.01
Creatinine B (mg/dl)	0.9	1.1	1.4	2.4	3.1	4.0	< 0.01
Operation (min)	227.0	270.0	325.0	275.0	335.0	425.0	< 0.01
Ischemia (min)	60.0	78.0	101.0	69.0	94.0	128.0	< 0.01
Perfusion (min)	97.0	124.0	156.0	124.0	168.0	220.0	< 0.01
Reperfusion (min)	27.0	37.0	48.0	36.0	50.0	74.0	< 0.01
Min. temp. (°C)	33.1	34.4	35.9	33.1	34.1	35.8	< 0.01

*means renal replacement therapy

Based on the median values, strongest differences became evident with respect to creatinine, with an increase of + 67% when measured immediately after surgery, or even of + 182% when measured 24 h post-surgery (both related to median values of patients with AKI, which were set to 100%). Median reperfusion and perfusion time were elevated by + 35%, operation and ischemia time by + 24% or + 21%, respectively (all: versus the 100% controls).

**Fig. 2:**
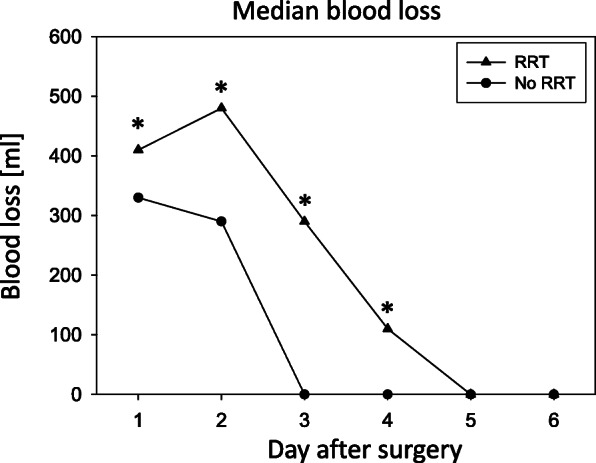
Median values of postoperative blood loss analyzed dynamically over 6 days post-operation

Postoperative blood loss was analyzed dynamically over 6 days and compared between those patients receiving hemofiltration and those patients without RRT. Figure 2 demonstrates that a greater blood loss was associated with a greater probability to develop AKI. Calculation of the longitudinal blood loss (day 1 - day 6) revealed a twofold increase of blood loss in AKI-patients versus those without AKI (1290 ml versus 620 ml). Strongest differences were seen after day 2 (+ 166% increase in patients with AKI, compared to patients without signs of AKI) and day 3 where no blood loss has been recorded at all in patients without AKI).

### Acute kidney injury defined by RIFLE

The incidence of AKI, defined according to the RIFLE criteria (increased creatinine × 3 or creatinine ≥ 4 mg/dl or 75% GFR), was 7% (*n* = 251 patients) and was, therefore, lower, compared to patients developing AKI based on the SOP criteria. Mortality was also lower in the RIFLE population (22%, *n* = 57 patients). Accordingly, risk factors were also different between SOP- and RIFLE-defined AKI. Most patients with normothermia and those receiving only cardiovascular bypass developed AKI (Table 3). Notably, valve surgery did not serve as a risk factor in the RIFLE cohort. The same was true with respect to the patients’ sex. On the other hand, the parameters re-operation, intra-aortic balloon pump were associated with AKI in both the SOP- and the RIFLE-cohort.

**Table 3:** Demographic and clinical parameters in the acute kidney injury (AKI) group according to the RIFLE-classification

Parameter	RRT*		*p*-value (chi-squared test)
	No	Yes	
	*n* = 3323	*n* = 251	
Sex			
Male	2341 (70%)	181 (72%)	
Female	981 (30%)	70 (28%)	0.62
Bypass surgery			
Yes	2520 (76%)	207 (82%)	
No	803 (24%)	44 (18%)	0.02
Valve surgery			
Yes	1157 (35%)	94 (37%)	
No	2166 (65%)	157 (63%)	0.10
Temperature			
Normothermia	2554 (76%)	177 (71%)	
Mild hypothermia	734 (22%)	63 (25%)	
Deep hypothermia	65 (2%)	11 (4%)	< 0.01
Surgery			
Bypass	2037 (61%)	153 (61%)	
Valve	596 (18%)	30 (12%)	
Bypass+valve	445 (13%)	50 (20%)	< 0.01

*means renal replacement therapy

### Clinical relevance of the creatinine serum level

The median and/or 25% or 75% quantile creatinine level was significantly higher in female patients and in patients receiving coronary artery bypass or valve replacement. Repeated surgical intervention was also coupled to an increased creatinine level, compared to patients with no previous operation. Patients with high serum creatinine were also more likely to have intra-aortic balloon pump use. Roller pump was superior to the centrifugal pump bypass in terms of a lower creatinine concentration. This was true with respect to the use of the Bretschneider solution (instead of blood cardioplegia).

Analysis of the metric parameters age, operation duration, ischemia time, minimum intraoperative body temperature, perfusion and reperfusion time and total blood loss, finally demonstrated a close association to the patients’ serum creatinine level.

## Discussions

AkI is associated with several complications as multiple organ dysfunction, systemic inflammation, and increased mortality. Based on a study on 50,314 adult surgical patients undergoing major inpatient surgery, the risk-adjusted average cost of care for patients undergoing surgery was $42,600 for patients with AKI compared to $26,700 for patients without AKI [8, 9].

Depending on the diagnostic criteria used to define AKI, the incidence of AKI following cardiovascular surgery ranges between 0.7–31%. This holds also true with respect to mortality rates which have been reported to vary between 30 and 90% [10]. The present study has been initiated to compare two different AKI-classification systems. The clinical SOP-criteria designated patients who were subjected to RRT following postoperative impairment of renal function as AKI positive. Patient data were then re-evaluated following the RIFLE-system, which includes serum creatinine and GFR, and AKI defined by this protocol.

Based on the SOP-definition, 10% of the patients developed AKI. Of these 48% died. The incidence of RRT was 10%, which is within the range reported by Kiers et al. (9.3%) [11]. Kristovic noted an incidence of 3.5% in a retrospective study, which is much lower than the values assessed in the present study [12]. Finally, from 31,677 patients who underwent open-heart surgery, 555 (1.7%) patients developed severe AKI requiring dialysis [13]. Presumably, the number of patients requiring RRT may at least partially depend on the patient cohort and kind of surgery.

Independent on this, 7% of the patients developed AKI according to the RIFLE classification with a mortality rate of 22%. The differences are remarkable. Obviously, integration of creatinine and GFR as risk parameters may reduce the number of patients classified into the AKI-group. Therefore, it is not surprising that mortality rates are also diminished compared to the one of patients who were subjected to hemofiltration (SOP-criteria). Similar to the present study, high AKI rates and mortality have been documented by others when AKI has been directly correlated to RRT. Based on Bahar et al., mortality of patients who developed postoperative AKI mandating hemodialysis was 79.9%, and Chertow et al. calculated the mortality of AKI patients sufficient to require dialysis to be 63.7% [14].

Since patients may develop AKI without requiring RRT, the AKI criteria has been widened by including elevation of serum creatinine levels or the reduction of urine output. Still, incidence and mortality of AKI strongly depends on the patient population and the final criteria included. The own AKI-evaluation was based on the RIFLE criteria with a threefold elevation of serum creatinine, or a maximum creatinine level of 4.0 mg/dl, or a decrease of the GFR by 75%.

Evaluation of patients after transcatheter aortic valve implantation by the RIFLE score revealed an AKI incidence of 17.9%, which is similar to the data given by Jorge-Monas on patients with cardiac surgery–associated AKI [15]. Incidence of AKI of patients undergoing elective, urgent or emergency cardiac surgery using the same scoring system was 25.1% [16].

In fact, 49.9% adult patients undergoing cardiac surgery, with or without cardiopulmonary bypass, developed postoperative AKI according to RIFLE, however, elevated serum creatinine was observed in only 9.7%, whereas oliguria was noted in 40.2% [17].

The different pathophysiologic significance of creatinine level and urine output might partially explain the heterogeneity of clinical studies. Indeed, the development of AKI is rather complex and depends on several parameters. AKI risk factors may closely depend on the patient cohort included in the study, the medical history and treatment practice. Based on the present work, a significant correlation has been found between the patient’s sex and AKI development, independent on whether the DOI or the RIFLE criteria has been applied. Gender associated AKI-incidence has also been reported by others with women being more likely than men to develop cardiac surgery-associated AKI postoperatively [18].

An observational study of 15,221 nondialysis-dependent patients undergoing cardiac surgery demonstrated an increased AKI-risk of males as well. The same was true with respect to AKI incidence after aortic valve replacement. In contrast, a retrospective single-centre cohort study of 565 consecutive patients who underwent isolated coronary artery bypass grafting with the use of cardiopulmonary bypass did not reveal any correlation between sex and AKI development [19], and Neugarten et al., analyzing sex differences in acute kidney injury requiring dialysis, even showed that male sex might be associated with an increased incidence of hospital-associated AKI [20]. Independent on the fact, that the references cited are based on different patient populations which may contribute to the contradictory statements, there is no general consensus that being a woman is an independent risk factor for the developing AKI. To optimize gender-associated risk assessment, a uniform scoring system is required which should additionally taken care on the type and severity of the illness and the type of surgical intervention. Although the own investigation did not reveal any relationship between gender and RRT, other reports did with nondialysis-dependent females but dialysis-dependent males being associated with AKI. This finding requires further evaluation.

Patients with an old age more often developed AKI than younger patients did. These alterations concern loss of renal mass, loss of tubules and sclerotic changes, which all may be accompanied by a decreased glomerular filtration rate and renal blood flow [21]. Screening healthy kidney donors have revealed a decline of GFR at a rate of 6.3 mL/min/1.73 m^2^ per decade. Consequently, due to the less kidney functional reserve, older people may be at higher risk for AKI. Not at least additional basic complaints which are more common in older people may account for the relationship between renal function and age. In good agreement with the own data, recent trials have identified old age as an independent factor associated with a higher risk for AKI and RRT failure [22].

Beside patient specific characteristics, several clinical risk factors have been identified to be associated with AKI. Patients requiring IABP more often received RRT than patients without IABP. The finding, which has also been shown by others, is interpreted in a way that IABP is used for critically ill patients with a low cardiac output. Since impairment of the cardiac output is directly attributed to hypoperfusion of the kidney and loss of kidney function, it might not be astonishing that patients with a pre-damaged kidney are highly predestinated for developing AKI. Interestingly, Zhang and coworkers provided evidence that preoperatively applied IABP may significantly reduce the incidence of AKI of high-risk patients [23]. A study on patients with acute myocardial infarction, severely impaired left ventricular ejection fraction or low output syndrome undergoing coronary surgery demonstrated a potent benefit of the preoperative compared to intraoperative IABP [24]. Nevertheless, whether preoperative IABP might provide any advantage for the patient cohort evaluated here is not clear. Further evaluation is necessary to investigate the risk-benefit balance of this strategy.

A high percentage of patients undergoing heart valve operations developed AKI. The relevance of valve surgery as a risk parameter became evident in both the SOP and the RIFLE cohort. This finding is notable, indicating that AKI in the context of valve replacement is not exclusively restricted to patients with RRT. Rather a high creatinine level may also linked to AKI in this matter. Indeed, data provided by others indicate that AKI, defined by either RIFLE, AKIN (Acute Kidney Injury Network) or KDIGO (Kidney Disease: Improving Global Outcomes), occurs in up to a quarter of patients following aortic valve replacement [25].

Haase et al. suggested that AKI may also reflect a form of pigment nephropathy with hemoglobin being the pathophysiologic key factor [26]. This postulate has recently been supported by others demonstrating distinct hemolysis and heme pigment deposition in the renal tubules following cardiopulmonary bypass. In good context, a case–control study of AKI patients provided evidence that patients who developed AKI had twice the plasma-free hemoglobin at the end of cardiopulmonary bypass than those who did not develop AKI, despite similar AKI risk profiles and identical cardiopulmonary bypass durations in each group. The authors reasoned that the pump of the CPB circuit along with the oxygenator, suction catheters, and filters damage erythrocytes and increase plasma-free hemoglobin which then may contribute to the development of AKI following cardiac surgery [27].

When interpreting the valve data, it must be considered that co-parameters may additionally influence the patients outcome. In this concern, a retrospective analysis of 7233 cardiac surgery patients showed that valve surgery combined with coronary artery bypass grafting might contribute to AKI more distinctly than each factor alone [18].

Further independent risk factors have been identified in the present evaluation: Previous coronary intervention, cross clamp time and duration of the surgical process. Mean value of surgical time was calculated to be 270 min. Yamauchi et al. recently pointed to an operation time longer than 8 h as an independent risk factor of postoperative AKI. However and in contrast to the own analysis, this study was enrolled on non-dialysis-dependent patients who underwent valvular operations using cardiopulmonary bypass. It is, therefore, concluded that AKI more often develops when the duration of cardiac surgery is prolonged. Still, there is no general rule, the critical time point rather depends on the kind of operation.

## Conclusions

In conclusion, several perioperative risk factors have been identified which are closely associated with AKI. These factors include long surgery time, prolonged ischemia time, distinct blood loss and hypothermia. This correlation became evident in the patient cohort subjected to RRT and in the cohort evaluated by the RIFLE criteria. From a clinical viewpoint, patients may not prone to AKI when surgery and ischemia time will be kept short, when blood loss is mitigated to a minimum and when surgery is performed under non-hypothermic conditions. Concerning the noxious effects free hemoglobin exerts on the kidney, clearing free hemoglobin from human plasma might be an attractive option to prevent renal damage.

Several trials and experimental models point to an important role of inflammation in the establishment of AKI.

To reduce the complication and mortality rate, patient surveillance, risk assessment and risk prevention should be considerably improved. Since the incidence of AKI is particularly high after major vascular surgery, an optimized consensus should take care on the type of surgical procedure performed. Discrimination between no- and high-risk patients might allow to manage patients without a risk factor in a perioperative fast-track manner, whereas the high-risk patients may be subjected to further tests, e.g. by analyzing novel risk-associated biomarkers. Not at least incorporation of real-time electronic kidney injury alerts in the latter patient cohort might be helpful to timely recognize the onset of AKI and to minimize the consequences associated with this disease.

## Data Availability

Yes.

## Abbreviations

AKI: Acute kidney injury
RRT: Renal replacement therapy
RIFLE: Risk, Injury Failure Loss End Stage
PRIFLE: Pediatric Risk Injury Failure Loss End Stage Renal Disease
GFR: Glomerular filtration rate
AkIN: Acute Kidney Injury Network
KDIGO: Kidney Disease: Improving Global Outcomes
CVVH: Continuous Veno-Venous Hemodialysis
SOP: Standard Operating Procedure
IABP: Intra aortic balloon pump
